# Age-specific Multimorbidity Patterns and Burden on All-Cause Mortality and Public Direct Medical Expenditure: A Retrospective Cohort Study

**DOI:** 10.1007/s44197-024-00256-y

**Published:** 2024-06-13

**Authors:** Sabrina Nan Hong, Francisco Tsz Tsun Lai, Boyuan Wang, Edmond Pui Hang Choi, Ian Chi Kei Wong, Cindy Lo Kuen Lam, Eric Yuk Fai Wan

**Affiliations:** 1 Department of Family Medicine and Primary Care, School of Clinical Medicine, Li Ka Shing Faculty of Medicine, The University of Hong Kong, Hong Kong SAR, China; 2 Centre for Safe Medication Practice and Research, Department of Pharmacology and Pharmacy, Li Ka Shing Faculty of Medicine, The University of Hong Kong, Hong Kong SAR, China; 3 Laboratory of Data Discovery for Health (D24H), Hong Kong Science and Technology Park, Hong Kong Science Park, Hong Kong SAR, China; 4 School of Nursing, Li Ka Shing Faculty of Medicine, The University of Hong Kong, Hong Kong SAR, China; 5 Department of Family Medicine, the University of Hong Kong Shenzhen Hospital, The University of Hong Kong, Hong Kong SAR, China; 6 Aston Pharmacy School, Aston University, Birmingham B4 7ET, United Kingdom; 7 School of Pharmacy, Medical Sciences Division, Macau University of Science and Technology, Macau, Macau SAR

**Keywords:** Multimorbidity, All-cause mortality, Healthcare expenditure, Observational study

## Abstract

**Objective:**

To evaluate age-specific multimorbidity patterns and morbidity burden on mortality and healthcare expenditure across age groups.

**Patients and Methods:**

Retrospective observational study between January 1, 2009 to December 31, 2017 using electronic health records in Hong Kong: Individuals were stratified by age (< 50, 50–64, 65–79, ≥ 80), and sub-classified by number of morbidities (0, 1, 2, 3, ≥ 4) out of 21 common chronic conditions. Clustering analyses were conducted to identify specific patterns of multimorbidity. Association between the number as well as combinations of morbidities and all-cause mortality and public expenditure was examined.

**Results:**

4,562,832 individuals with a median follow-up of 7 years were included. Mental disorders were the top morbidities among young individuals, while cardiovascular diseases were prevalent in the elderly. An increased number of morbidities was associated with a greater relative risk for mortality and medical expenditure, and this relationship was stronger among younger patients. Compared to individuals in the same age group without morbidity, the hazard ratios (HR; 95% CI) of all-cause mortality in patients aged < 50 and ≥ 80 with two comorbidities 3.81 (3.60–4.03) and 1.38 (1.36–1.40), respectively, which increased to 14.22 (9.87–20.47) and 2.20 (2.13–2.26), respectively, as the number of morbidities increased to ≥ 4. The stroke-hypertension cluster was shown to be associated with the highest HR of mortality 2.48 (2.43–2.53) among all identified clusters arising from the clustering analysis.

**Conclusion:**

Given the stronger association between multimorbidity and all-cause mortality and greater opportunity costs in younger populations, prevention and management of early-onset multimorbidity are warranted. (248 words)

**Supplementary Information:**

The online version contains supplementary material available at 10.1007/s44197-024-00256-y.

## Introduction

Multimorbidity, defined as the coexistence of two or more chronic diseases [1], is consistently shown to be associated with an increased risk of mortality [2], healthcare utilization [3], and economic burden [4], for both individuals and society as a whole. Although multimorbidity mainly affects older people [5], recent studies suggested it has a notable increased prevalence among young individuals [6, 7], which could be attributed to increased healthcare awareness [8], physical inactivity, alcohol abuse, and an unhealthy diet [9].

Differences in the association of multimorbidity with mortality among individuals of different ages have only been examined to a minimal extent. Previous research based on general population largely support a stronger association of multimorbidity with subsequent adverse health outcomes [10]. To date, however, existing studies focused on comparing differences in strength of such associations between individuals of different ages are either limited by a modest sample size [11] or with a short follow-up period [12]; while others are primarily focused on the older population [13, 14]. In addition, although a significant number of studies have investigated the individual relationships between different common chronic diseases [15–17], they have primarily focused on the links between specific diseases rather than including a broad range of conditions to investigate a broad picture of multimorbidity patterns and the associated mortality risks. Also, data from young adult populations remain scant in this regard, as reflected by the predominance of elderly-based studies in a systematic review which aims to identify multimorbidity patterns [18]. Therefore, a comprehensive evaluation of multimorbidity across all age ranges with a large population-based electronic health records database with a sufficient longitudinal follow-up period is much warranted to inform age-specific clinical management and primary care policies.

In this study, using a public healthcare database in Hong Kong which covers the vast majority of the population with records spanning over two decades, we aim to evaluate the age-specific relationship between multimorbidity and all-cause mortality and public direct medical expenditure over a period of 10 years. The second objective of this study is to investigate age-specific multimorbidity patterns among the general population of Hong Kong.

## Methods

### Study Design

This was a retrospective observational study based on electronic medical records from the Hong Kong Hospital Authority (HA), which includes comprehensive health documentation of the local population, over 90% of which are of Chinese ethnicity [19]. HA, the statutory body of public healthcare service providers in Hong Kong, is constituted of 43 public hospitals and institutions, 49 specialist outpatient clinics and 73 outpatient primary care clinics, under which all the healthcare professionals have been well-trained in using the electronic medical records to document clinical information of every patient. Individuals aged 18 years or above with medical records in the HA electronic medical record system between January 1st 2009 to 31st December 2017 were included in this study. They were then classified into five groups according to their number of morbidities (0, 1, 2, 3, and ≥ 4). The baseline date was defined as the first date of doctor consultation attendance in this period, and all patients were followed until mortality or 31st December 2018, whichever occurred earlier.

### Definition of Morbidity

A total of 21 chronic conditions proved to be of high validity for multimorbidity studies were included in this study [20], and ICPC-2 and ICD-9-CM adopted in this reference paper were also used to define the conditions in this study. The ICPC-2 and ICD-10 codes are detailed in supplementary Table 1. Conditions that are not coded in the local electronic medical records (Chronic viral hepatitis B, Epilepsy, Inflammatory bowel disease, Irritable bowel syndrome, Multiple sclerosis, Parkinson’s disease, and Psoriasis) were excluded. Due to the low completeness of coding for chronic kidney disease (CKD), an estimated glomerular filtration rate (eGFR) of < 60 ml/min/1.73m^2^ was additionally used to define CKD as suggested by other studies [21].

### Outcome Measurement

The primary outcome of this study was all-cause mortality. Mortality data was sourced from the Hong Kong Deaths Registry, which is a governmental body that records the mortality of Hong Kong residents. The secondary outcome was the annual public direct medical costs, which were calculated by multiplying the number of medical attendances with their respective costs, including those arising from attendances at the general ward, Accident & Emergency Department, specialist clinic, general clinic, and allied health. The unit cost for relevant health service utilisation, which includes consultation, investigations, medications and other treatments, was published by the Government of the Hong Kong Special Administrative Region Gazette and Hospital Authority Ordinance (Chap. 113) in 2013 and listed in supplementary Table 2 [22]. Aside from the two main outcomes, this study also aims to provide additional information regarding the trend of multimorbidity prevalence from 2009 to 2017. Individuals meeting the inclusion criteria (aged 18 years or above with medical records in the HA electronic medical record system between January 1st 2009 31st December 2017) with medical attendance in any year were included in the calculation of the prevalence of multimorbidity in the corresponding year.

### Statistical Analysis

Descriptive statistics were used to summarize age and sex distribution. Chord diagrams were used to illustrate the morbidity combination patterns. Kaplan-Meier curves were plotted to display the survival probability by number of conditions overall and in different age groups. K-means clustering was used to identify clusters of patients having various non-random combinations of diseases with the elbow point method used to determine the number of clusters [23]. Cox proportional hazard regression adjusting for age and sex was used to demonstrate the association between the number and combinations of morbidities and all-cause mortality, and hazard ratios (HRs) were used to represent the mortality risk. To account for the highly positively skewed distribution of cost data, gamma generalised linear model with log link function adjusting for age and sex was used to examine the association between the number of morbidities and annual public direct medical costs [24, 25]. Relative risks (RRs) for increased healthcare costs were used for comparison between different groups. Patients were further stratified into four age groups (< 50, 50–64, 65–79, ≥ 80 years), and the analysis was repeated to analyse the patterns and burden of multimorbidity on individuals of different age groups. All significant tests were two-tailed and rejected its null hypothesis at a *p*-value less than 0.05. Statistical analyses were performed using Stata version 16.0 (College Station, Texas).

## Results

**Fig. 1:**
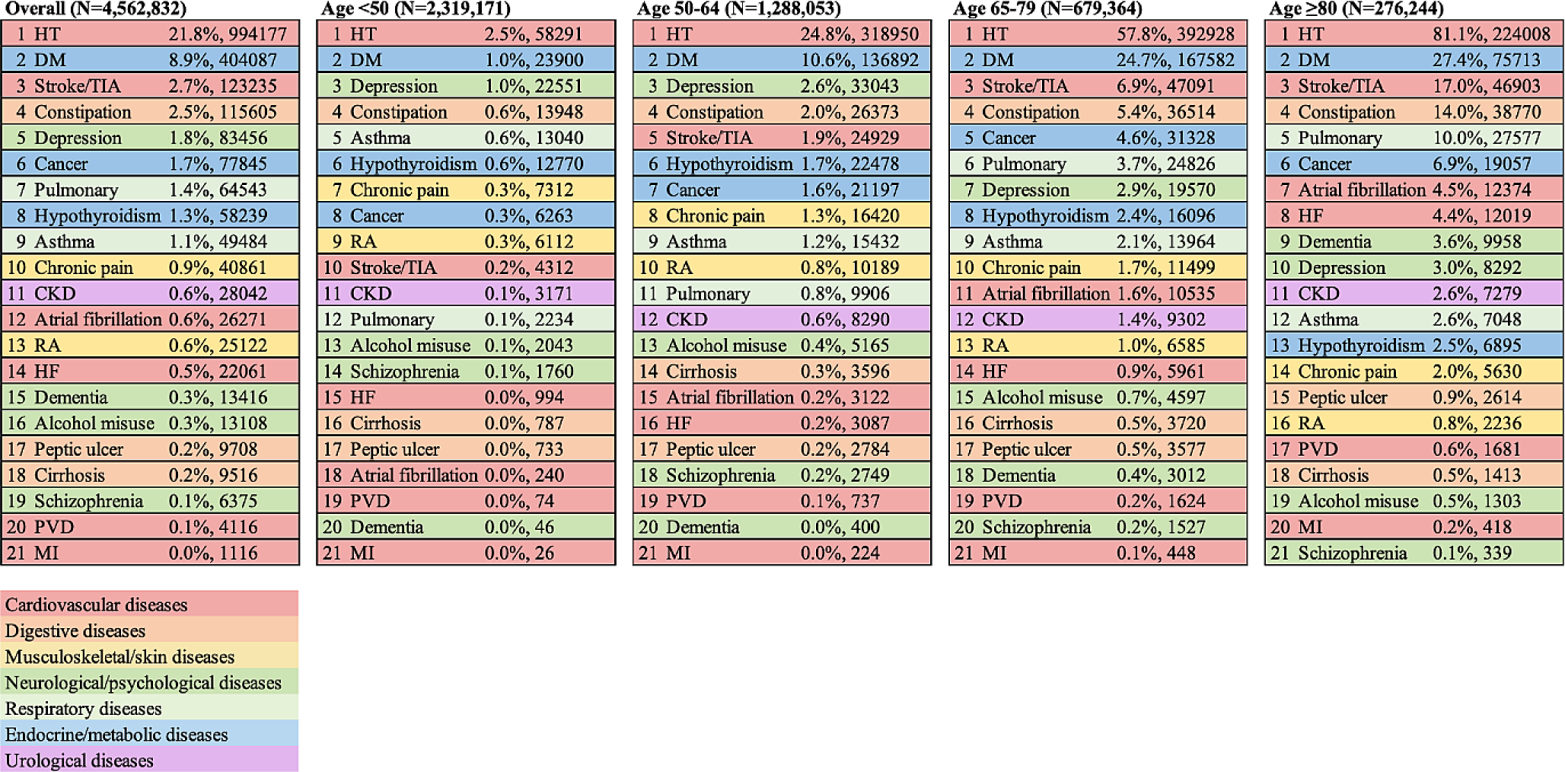
Prevalence of individual morbidities in the overall cohort and different age groups. HT: Hypertension; DM: Diabetes; Stroke/TIA: Stroke or transient ischemic attack; Constipation: Severe constipation; Pulmonary: Chronic Pulmonary disease; CKD: Chronic kidney disease; RA: Rheumatoid arthritis; HF: Chronic heart failure; PVD: Peripheral vascular disease; MI: Myocardial infarction.

A total of 4,562,832 individuals over a median follow-up of 7 years (85.5 months) were included in this study. The mean age was 49.4 years (SD 18.0), and 45.9% were males. Table 1 shows the baseline characteristics for individuals with different numbers of morbidities for all patients and stratified by age groups. Overall, 9.1% of the study cohort had two or more chronic morbidities. The prevalence of multimorbidity increased with age, ranging from 1.0% in individuals aged < 50, up to 31.8% in those aged ≥ 80. From Fig. 1 showing the trend of morbidity count from 2009 to 2018, the prevalence of multimorbidity increased over the past 9 years, from 14.0% in 2009, to 18.9% in 2013, and 24.1% in 2018. Detailed percentages of individuals with different number of morbidities across these years were listed in supplementary Table 3.

**Table 1:** Baseline characteristics of patients with different number of morbidities in different age groups

	Overall	Age, years			
		< 50	50–64	65–79	≥ 80
N	4,562,832	2,319,171	1,288,053	679,364	276,244
**Sex**					
Female	2,469,714 (54.1%)	1,307,912 (54.1%)	668,114 (51.9%)	327,336 (48.2%)	166,352 (60.2%)
Male	2,093,118 (45.9%)	1,011,259 (45.9%)	619,939 (48.1%)	352,028 (51.8%)	109,892 (39.8%)
**Number of conditions**					
0	3,341,075 (73.2%)	2,122,864 (91.5%)	844,896 (65.6%)	286,707 (42.2%)	86,608 (31.4%)
1	855,968 (18.8%)	173,276 (7.5%)	338,396 (26.3%)	242,566 (35.7%)	101,730 (36.8%)
2	297,343 (6.5%)	21,335 (0.9%)	93,214 (7.2%)	120,045 (17.7%)	62,749 (22.7%)
3	57,041 (1.3%)	1,571 (0.1%)	10,425 (0.8%)	25,365 (3.7%)	19,680 (7.1%)
≥ 4	11,405 (1.3%)	125 (0.0%)	1,122 (0.1%)	4,681 (0.7%)	5,477 (2.0%)

All parameters are expressed in mean (standard deviation) or frequency (percentage)

Table 1 shows the baseline characteristics of individuals with different numbers of morbidities, further stratified by age groups. Overall, 9.1% of the study cohort had two or more chronic morbidities. The prevalence of multimorbidity increased with age, ranging from 1% in individuals aged < 50, 8.1% in 50–64 years, 22.1% in 65–79 years, and up to 31.8% in ≥ 80 years.

**Fig. 2:**
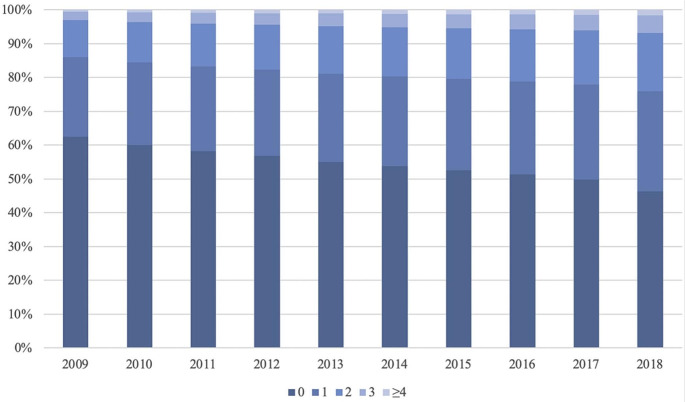
Trend of morbidity count from 2009 to 2018

Figure 2 shows the prevalence of individual morbidities in the overall cohort and different age groups in 2018. The most prevalent morbidity overall was hypertension (HT; 21.8%), followed by diabetes (DM; 8.9%) and stroke or transient ischemic attack (2.7%). The prevalence of most chronic conditions increased with age, and diseases with the most drastic increase in prevalence from individuals aged < 50 to those aged ≥ 80 included HT (from 2.5 to 81.1%), diabetes (from 1.0 to 27.4%), and stroke/transient ischemic attack (from 0.2 to 17.0%).

Supplementary Fig. 1 shows the chord diagrams illustrating the relative frequencies of morbidity combinations of the five most prevalent diseases in each age group. Only the top five diseases in each group were selected given the low prevalence of the remaining diseases which might make their relationship with other conditions less generalisable. Cardiovascular diseases (CVDs; conditions A-F) were frequent morbidities that co-occur with the top five diseases in each age group, constituting more than half of all possible combinations. A detailed description of the top 10 morbidity combinations in different age groups was listed in supplementary Table 4. The combination of HT plus DM was the most common morbidity pattern across all age groups, and all top combinations included at least one of them. Given the dominating effect of HT, the analysis was repeated by excluding this condition, and the results were shown in supplementary Table 5. DM remained a frequent combination component in all age groups; for age-specific conditions, psychological conditions such as depression and alcohol misuse were frequent among younger patients, while CVDs such as stroke or transient ischemic attack (TIA), chronic pulmonary disease, and constipation, had emerged as increasingly prevalent morbidities as age increased.

**Fig. 3:**
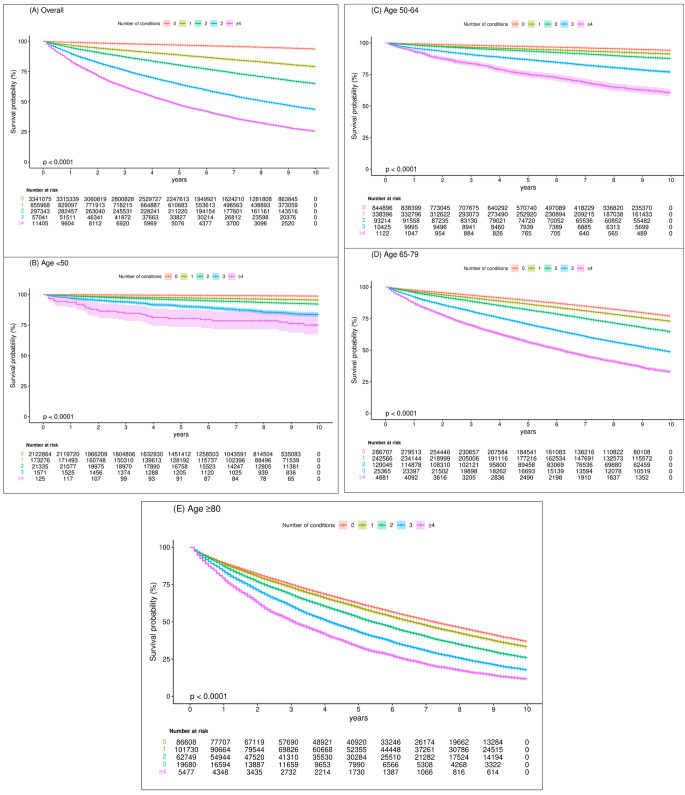
Kaplan-Meier curves of all-cause mortality in groups with different numbers of conditions overall and in different age groups

**Fig. 4:**
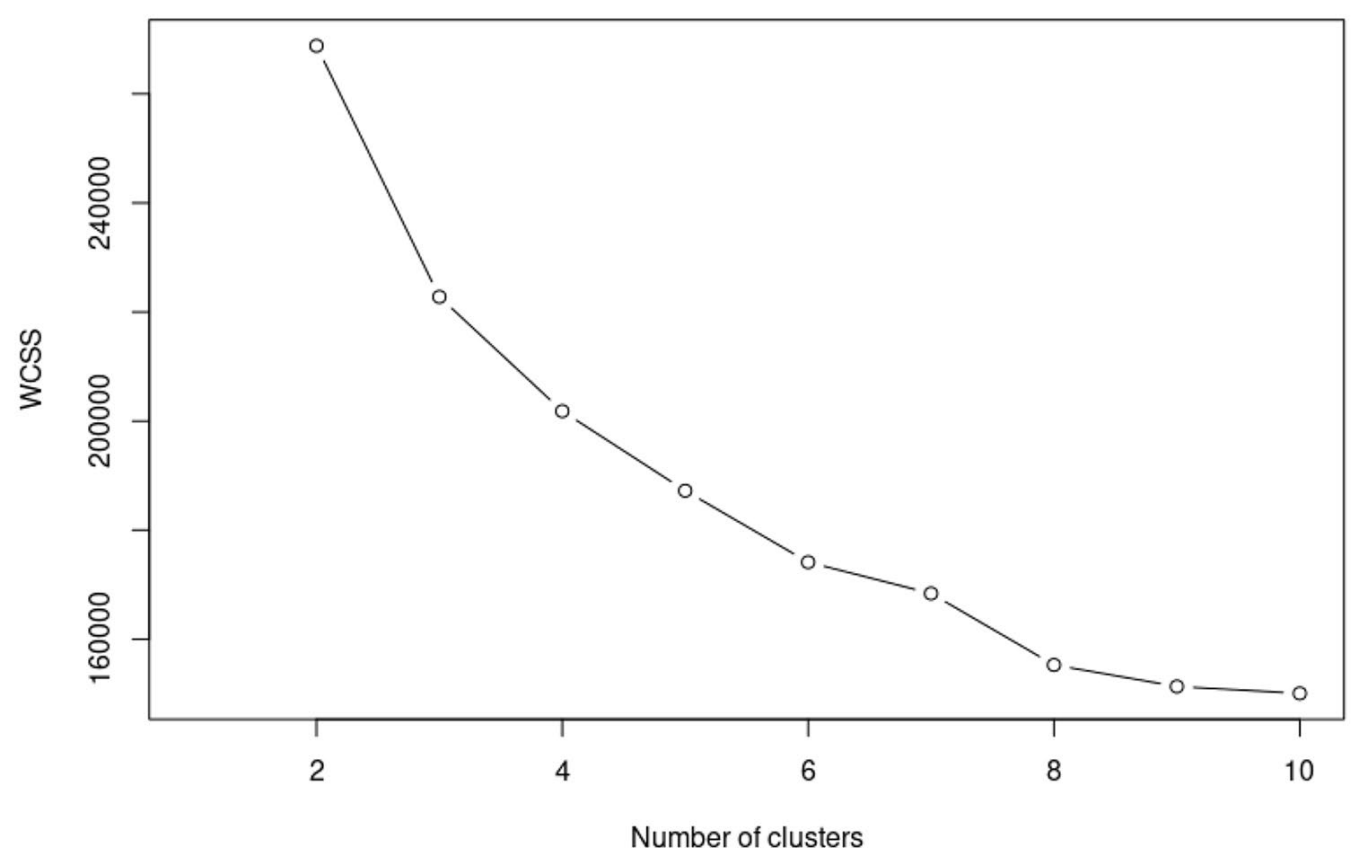
Within-cluster sum of squares across various numbers of clusters in K-means clustering WCSS = Within-Cluster Sum of Squares

Table 2; Fig. 3 show the Kaplan–Meier survival curves, 10-year absolute risk difference (ARD) and HRs for all-cause mortality, and RRs for an increase in annual direct public medical costs among individuals with different numbers of morbidities in the overall cohort, further stratified by age groups. Compared to individuals without any morbidities, incidence rates and risks for all-cause mortality increased in individuals with a greater number of morbidities, with the corresponding HRs (95% CI) being 1.25 (1.24–1.26, *p* <.001) for one condition, and up to 2.92 (2.85–2.99, *p* <.001) for four or more conditions. Similarly, the ARD for all-cause mortality increased from 1.52 (1.46, 1.58) for one condition, to 4.16 (3.50, 4.49) for four or more conditions. Comparing individuals across age groups, the incidence rates for mortality increased with age, and HRs of those aged < 50 were the highest, with larger HRs at any given number of morbidities compared to patients of older age with the same morbidity count. The increase in the number of morbidities had a larger effect on all-cause mortality in younger individuals, as shown by the greater increase in HRs as well as ARDs for every increase in the number of conditions in the younger age groups Consistent with the observations for all-cause mortality, a greater number of morbidities was also associated with greater public direct medical costs. Compared to individuals without any morbidities, the RRs for annual public direct medical cost increased in patients with a greater number of morbidities, with the corresponding RRs (95% CI) being 1.85 (1.82–1.87, *p* <.001) for one condition, and up to 4.66 (4.21–5.16, *p* <.001) for four or more conditions. The relative increase in medical costs with a greater number of morbidities was more significant in the younger age groups, given that RR among patients < 50 years increased from 2.81 to 19.7 as the number of morbidities increased from 1 to ≥ 4, which was more substantial relative to those in the ≥ 80 age group (from 1.19 to 2.16). Table 3 shows the baseline characteristics stratified by clusters of patients with various combinations of diseases arising from the K-means clustering analysis. The elbow point method determined that 8 clusters existed (Fig. 4). Table 4 shows the age- and sex-adjusted HR associated with each cluster compared with Cluster 1. Notably, the stroke-hypertension cluster (Cluster 3, HR[95% CI] 2.48 [2.43–2.53]) and the diabetes-hypertension cluster (Cluster 5, HR [95% CI] 2.21 [2.16–2.25]) were associated with substantially higher mortality rates.

**Table 2:** Incidence and incidence rate of all-cause mortality in groups with different number of conditions in different age groups

**Overall**						
No. of conditions	Event (*N* = 409,191)	Incidence Rate	ARD (95% CI)	HR	Annual Total Cost (USD)	RR
0	136,733 (4.09%)	6.372 (6.34, 6.41)			1629.37	
1	143,099 (16.72%)	23.869 (23.75, 23.99)	1.52 (1.46, 1.58)	1.25 (1.24,1.26)	5185.60	1.85 (1.82,1.87)
2	91,737 (30.85%)	43.622 (43.34, 43.91)	3.44 (3.38, 3.56)	1.57 (1.56,1.59)	8240.22	2.24 (2.19,2.29)
3	29,592 (51.88%)	85.288 (84.32, 86.27)	7.15 (6.98, 7.32)	2.21 (2.18,2.24)	14107.05	3.38 (3.22,3.53)
≥ 4	8030 (70.41%)	144.196 (141.08, 147.39)	11.08 (10.7, 11.46)	2.92 (2.85,2.99)	21373.37	4.66 (4.21,5.16)
**Age < 50**						
No. of conditions	Event (*N* = 24,820)	Incidence Rate	ARD (95% CI)	HR	Annual Total Cost (USD)	RR
0	17,103 (0.81%)	1.244 (1.23, 1.26)			780.04	
1	6100 (3.52%)	4.950 (4.83, 5.08)	2.12 (2.02, 2.22)	2.71 (2.63,2.79)	2483.95	2.81 (2.72,2.90)
2	1368 (6.41%)	8.395 (7.96, 8.85)	3.46 (3.2, 3.72)	3.81 (3.60,4.03)	3752.84	4.02 (3.68,4.39)
3	220 (14.00%)	18.587 (16.29, 21.21)	8.72 (7.52, 10.06)	8.29 (7.25,9.47)	8359.91	8.78 (6.36,12.12)
≥ 4	29 (23.20%)	31.966 (22.21, 46.00)	15.24 (10.5, 21.6)	14.22 (9.87,20.47)	19449.87	19.70 (6.28,61.73)
**Age 50–64**						
No. of conditions	Event (*N* = 69,460)	Incidence Rate	ARD (95% CI)	HR	Annual Total Cost (USD)	RR
0	33,360 (3.95%)	6.090 (6.03, 6.16)			1798.07	
1	23,702 (7.00%)	9.586 (9.46, 9.71)	2.74 (2.63, 2.91)	1.48 (1.46,1.51)	3236.69	1.74 (1.71,1.78)
2	9874 (10.59%)	13.465 (13.20, 13.73)	5.35 (5.08, 5.57)	1.95 (1.90,1.99)	4172.59	2.17 (2.09,2.24)
3	2125 (20.38%)	27.156 (26.03, 28.34)	14.52 (13.73, 15.35)	3.72 (3.56,3.89)	7891.37	3.98 (3.62,4.38)
≥ 4	399 (35.56%)	52.576 (47.66, 58.00)	28.96 (26.29, 31.82)	6.97 (6.32,7.70)	14380.74	7.04 (5.26,9.42)
**Age 65–79**						
No. of conditions	Event (*N* = 151,641)	Incidence Rate	ARD (95% CI)	HR	Annual Total Cost (USD)	RR
0	44,448 (15.50%)	24.655 (24.43, 24.89)			4347.48	
1	54,312 (22.39%)	31.158 (30.90, 31.42)	3.55 (3.35, 3.93)	1.18 (1.17,1.20)	6231.05	1.38 (1.35,1.40)
2	37,918 (31.59%)	42.911 (42.48, 43.35)	10.68 (10.33, 11.03)	1.57 (1.55,1.59)	7972.43	1.72 (1.69,1.76)
3	12,000 (47.31%)	71.797 (70.52, 73.09)	23.92 (23.22, 24.61)	2.42 (2.37,2.47)	12633.50	2.63 (2.52,2.74)
≥ 4	2963 (63.30%)	113.747 (109.72, 117.92)	37.93 (36.58, 39.24)	3.59 (3.46,3.72)	19240.12	3.90 (3.55,4.29)
**Age ≥ 80**						
No. of conditions	Event (*N* = 163,270)	Incidence Rate	ARD (95% CI)	HR	Annual Total Cost (USD)	RR
0	41,822 (48.29%)	97.279 (96.35, 98.22)			11803.61	
1	58,985 (57.98%)	107.778 (106.91, 108.65)	4.16 (3.5, 4.49)	1.12 (1.10,1.13)	13777.37	1.19 (1.16,1.21)
2	42,577 (67.85%)	131.781 (130.53, 133.04)	11.63 (11.13, 12.13)	1.38 (1.36,1.40)	16320.76	1.43 (1.40,1.47)
3	15,247 (77.47%)	169.899 (167.22, 172.62)	19.42 (18.71, 19.94)	1.75 (1.71,1.78)	19757.66	1.74 (1.68,1.80)
≥ 4	4639 (84.70%)	219.415 (213.19, 225.82)	25.74 (24.93, 26.38)	2.20 (2.13,2.26)	24672.96	2.16 (2.04,2.29)

All parameters are expressed in frequency (percentage). Incidence rate (cases/1000 person-years) with 95% confidence interval based on Poisson distribution. Absolute risk difference (ARD), hazard ratio (HR) and relative risk (RR) were adjusted for baseline characteristics including age and sex

**Table 3:** Baseline characteristics of patients by cluster

Name	Cluster								SMD
	1	2	3	4	5	6	7	8	
Number of subjects	18,172	28,443	61,416	46,792	166,417	10,078	19,126	15,345	0
Age, years (mean (SD))	61.40 (14.85)	76.87 (10.52)	72.70 (12.58)	71.89 (13.28)	66.87 (11.53)	77.70 (10.22)	73.20 (10.38)	65.45 (13.32)	0.557
Sex, male (%)	8686 (47.8)	19,674 (69.2)	34,561 (56.3)	21,812 (46.6)	74,590 (44.8)	4950 (49.1)	9856 (51.5)	3231 (21.1)	0.314
Charlson Comorbidity Index (mean (SD))	1.87 (2.20)	2.44 (1.89)	2.49 (1.78)	2.02 (2.24)	0.86 (1.41)	1.58 (1.69)	3.06 (1.91)	0.65 (1.31)	0.567
***Chronic conditions (%)***									
Hypertension	0 (0.0)	*21,440 (75.4)*	*57,837 (94.2)*	**46,792 (100.0)**	**166,417 (100.0)**	*8461 (84.0)*	*16,834 (88.0)*	*14,843 (96.7)*	NA
Diabetes	*9167 (50.4)*	*3106 (10.9)*	0 (0.0)	0 (0.0)	**166,417 (100.0)**	1826 (18.1)	**19,126 (100.0)**	56 (0.4)	NA
Stroke or transient ischemic attack	135 (0.7)	1790 (6.3)	**61,416 (100.0)**	0 (0.0)	0 (0.0)	1800 (17.9)	**19,126 (100.0)**	39 (0.3)	NA
Severe constipation	**4439 (24.4)**	2189 (7.7)	*2666 (4.3)*	*11,252 (24.0)*	*3799 (2.3)*	521 (5.2)	872 (4.6)	546 (3.6)	0.305
Depression	*3878 (21.3)*	955 (3.4)	2297 (3.7)	13 (0.0)	2084 (1.3)	183 (1.8)	525 (2.7)	**8101 (52.8)**	0.524
Cancer	3594 (19.8)	2500 (8.8)	1883 (3.1)	**11,185 (23.9)**	3336 (2.0)	333 (3.3)	488 (2.6)	324 (2.1)	0.313
Chronic pulmonary disease	1203 (6.6)	**28,443 (100.0)**	2334 (3.8)	0 (0.0)	0 (0.0)	764 (7.6)	686 (3.6)	23 (0.1)	NA
Hypothyroidism	2247 (12.4)	491 (1.7)	657 (1.1)	11 (0.0)	1898 (1.1)	418 (4.1)	281 (1.5)	**7990 (52.1)**	0.489
Asthma	**2319 (12.8)**	2509 (8.8)	516 (0.8)	5660 (12.1)	1800 (1.1)	211 (2.1)	218 (1.1)	305 (2.0)	0.249
Chronic pain	**1104 (6.1)**	204 (0.7)	260 (0.4)	1747 (3.7)	538 (0.3)	42 (0.4)	61 (0.3)	92 (0.6)	0.136
Chronic kidney disease	711 (3.9)	377 (1.3)	736 (1.2)	**3739 (8.0)**	1176 (0.7)	114 (1.1)	224 (1.2)	116 (0.8)	0.135
Atrial fibrillation	434 (2.4)	343 (1.2)	0 (0.0)	0 (0.0)	0 (0.0)	**10,078 (100.0)**	587 (3.1)	12 (0.1)	NA
Rheumatoid arthritis;	**956 (5.3)**	439 (1.5)	395 (0.6)	2050 (4.4)	244 (0.1)	32 (0.3)	41 (0.2)	95 (0.6)	0.153
Chronic heart failure	788 (4.3)	1852 (6.5)	1302 (2.1)	5857 (12.5)	1611 (1.0)	**1832 (18.2)**	430 (2.2)	195 (1.3)	0.272
Dementia	695 (3.8)	400 (1.4)	1600 (2.6)	**2363 (5.1)**	829 (0.5)	263 (2.6)	507 (2.7)	135 (0.9)	0.119
Alcohol misuse	**2723 (15.0)**	654 (2.3)	818 (1.3)	2467 (5.3)	634 (0.4)	102 (1.0)	145 (0.8)	77 (0.5)	0.220
Peptic ulcer disease	508 (2.8)	272 (1.0)	411 (0.7)	**1458 (3.1)**	484 (0.3)	133 (1.3)	141 (0.7)	72 (0.5)	0.100
Cirrhosis	**2211 (12.2)**	337 (1.2)	339 (0.6)	1108 (2.4)	311 (0.2)	29 (0.3)	52 (0.3)	29 (0.2)	0.185
Schizophrenia	**796 (4.4)**	81 (0.3)	95 (0.2)	636 (1.4)	498 (0.3)	9 (0.1)	54 (0.3)	31 (0.2)	0.107
Peripheral vascular disease	153 (0.8)	115 (0.4)	177 (0.3)	**687 (1.5)**	396 (0.2)	51 (0.5)	88 (0.5)	23 (0.1)	0.058
Myocardial infarction	126 (0.7)	71 (0.2)	70 (0.1)	**361 (0.8)**	165 (0.1)	29 (0.3)	28 (0.1)	9 (0.1)	0.052

Bold font denotes the cluster in which the corresponding disease is most prevalent (compared with all other clusters). Underscore denotes the most prevalent three diseases within each cluster. NA = not pplicable; SD = standard deviation; SMD = standardized mean difference.

**Table 4:** Risk of all-cause mortality by cluster

Cluster	Events	Follow-up time (years)	Incidence rate (95% CI) (per 1000 person years)	Hazard ratio (95% CI)
1	39,305	1,296,905	30.31 (30.01–30.61)	REF
2	26,406	326,055	80.99 (80.01–81.97)	1.78 (1.76–1.81)
3	11,602	109,426	106.03 (104.11-107.97)	2.48 (2.43–2.53)
4	6,203	124,755	49.72 (48.49–50.97)	1.89 (1.84–1.94)
5	16,506	123,148	134.03 (132.00-136.09)	2.21 (2.16–2.25)
6	9,436	115,204	81.91 (80.26–83.58)	1.89 (1.85–1.93)
7	14,070	290,301	48.47 (47.67–49.27)	1.19 (1.17–1.22)
8	5,831	85,511	68.19 (66.45–69.96)	1.74 (1.69–1.79)

Hazard ratio was adjusted with age and sex. REF **=** reference group; CI = confidence interval

## Discussion

In this study based on a large territory-wide cohort, a constantly high prevalence of multimorbidity over the past ten years was observed. The relationship between an increased number of morbidities and a greater relative risk for all-cause mortality and healthcare cost was demonstrated to be notably stronger among younger patients. Overall, HT was the most prevalent morbidity among patients of all age groups. Clustering analysis and subsequent Cox regression also showed that TIA + HT and DM + HT clusters were related to a higher risk of mortality. Following HT, after age group stratifications, CVDs such as DM and TIA were common among the older age group. Together with HT, these three diseases were the top 3 morbidities in the 50–64, 65–79, and ≥ 80 age groups. In contrast, in the younger age group (< 50 years), depression appeared to be a significant health condition following HT and DM.

Comparing the prevalence of multimorbidity in 2008 and 2017, the number has almost doubled from 14.0% in 2008 to 24.1% in 2017. Likewise, a Chinese study also found that the number of patients with two or more chronic diseases increased significantly from 32.5% in 2004 to 53.2% in 2017 [26, 27]. In the US, a sharp rise of patients with more than two morbidities from 45.7% in 1988 to 59.6% in 2014 has also been reported [28]. Various explanations have been suggested to account for this change, including the longer life expectancy creating an aging society with more elderly, who were the most vulnerable group to multimorbidity [29], increased public awareness of chronic diseases leading to earlier disease detection [27], unhealthy trends such as physical inactivity [30], and childhood obesity epidemic starting from the late 1950s [31]. In terms of demographics, an increased number of morbidities is associated with increased age. In this study, the proportion of individuals with multimorbidity (i.e., ≥ 2 morbidities) increased from 0.7% in the < 50 age group to 18.6% in the 65–79 age group. These numbers are similar to a study conducted in the Netherlands, which reported that 15% of individuals aged 65 or above in the general population were diagnosed with multimorbidity [32]. The relationship between age and the number of chronic conditions has been well-documented in [33].

The association between multimorbidity and a higher risk of all-cause mortality and more significant public direct medical expenditure has been well-established [34–37]. With age stratifications, this study additionally demonstrated that these associations were stronger in the younger population, reflected by their larger HRs at any given number of morbidities compared to patients of older age. With a more significant reduction in life expectancy per increase in the number of morbidities in the younger age group, it is suggested that an early presence and onset of multimorbidity is associated with increased mortality and burden to the healthcare system. In addition, the increases in mortality risk and medical cost per additional morbidity were of a larger magnitude in younger individuals, implying that the effect of multimorbidity increases with a younger age of multimorbidity diagnosis. Hence, early prevention is needed to avoid the development of multimorbidity at a young age to ward off the more devastating complications and higher healthcare costs that arise. Beyond healthcare costs, multimorbidity could also contribute to social costs, especially among the younger population which are the primary working force. Patients with multimorbidity are prone to functional limitations and more of their time is spent on healthcare visits, interfering with their work and productivity of the society as a whole [38]. Thus, the burden of multimorbidity among the younger working population, which extends from healthcare to social costs, is more significant to the economy.

Within the younger age group, in the < 50 age group, depression was the third most frequent morbidity, following HT and DM. Depression + HT and depression + DM were also the most common morbidity pair following HT + DM in this population. The prevalence of depression in young adults has surged over the past few years [39]. Studies reported that depression, attributable to a spectrum of factors such as neuroticism, early adversity and substance misuse, affected 25% of young adults [40]. From the multimorbidity aspect, depression is suggested to be associated with not only HT but also many other CVDs, supported by studies which demonstrated significant overlap between patients with depression and CVDs [41–43]. On top of it, there is a positive relationship between the severity of depression and the risk of CVDs and mortality [44]. A longitudinal study predicted a significant 70% increase in the risk of myocardial infarction among depressive patients [45], with several plausible mechanisms proposed, including autonomic nervous system alteration [46], platelet receptors and function abnormalities [47], and change in neurohormonal factors [48]. Given the close relationship between depression and HT, it has been suggested that depression should be considered during the treatment planning process for HT to improve the overall health outcome [49]. Concerning the DM + depression combination, it has been reported that these two diseases co-exist together twice as frequently as would be predicted by chance alone, with underlying hypotheses such as their shared underlying biological and behavioural mechanisms [50]. Given the high correlation of depression with various types of diseases, and that depression management has been demonstrated to mitigate the combined effect of multimorbidity and depression on mortality [51], it is crucial to integrate depression control strategies into the management plans of young individuals to improve the disease outcome of these multimorbid patients.

In contrast, TIA appeared to be a more significant burden in the older age groups compared to the younger cohort, given that TIA + HT and TIA + DM were the most frequent morbidity pair following HT + DM in this population. HT and DM are well-known common risk factors for CVDs [52, 53]; worse, patients with HT + DM are at an even higher risk of further CVDs [54]. In fact, the TIA + HT and HT + DM clusters were shown in our data to entail the highest risk of mortality, which is highly consistent with previous research on the association of specific multimorbidity patterns and mortality showing similar elevated risks for those people [55]. Given that HT and DM were also the most common diseases among the younger individuals, these close relationships between HT, DM and TIA and their markedly stronger association with mortality suggested that it is of paramount importance to control and prevent the occurrence of these two diseases at a young age to reduce the risk of developing devastating CVDs along the disease course.

This study has several strengths. With the use of a population-wide medical record system with documentation of more than half of the local population [56], this study is of high representativeness and generalizability. By repeating the analysis with age group stratifications and further use of K-means clustering analysis, a more comprehensive and detailed multimorbidity picture among individuals was demonstrated. Nonetheless, there were still several limitations. First, given that this is an observational study, a causal relationship between the number of comorbidities and all-cause mortality and healthcare costs could not be established. Second, the medical record system adopted was at the public healthcare level, thus, it might not apply to those seeking medical care in the private healthcare sector. Previous study depicted that chronic diseases were more commonly encountered in the public healthcare sector [57]; hence, the study results might not accurately represent the multimorbidity prevalences and patterns in the private healthcare system. Given that the HA service utilization rate is low among younger individuals and has only increased exponentially for people aged 65 years or above, there is also possible selection bias [58]. Third, as it is for studies based on electronic medical records, other possible confounders, such as socio-economic background and lifestyle factors, which are related to an individual’s health, were not comprehensively addressed due to the lack of data in the database. Also, possible under-documentations on the medical system might affect the accuracy in reflecting the actual disease prevalences. Last, morbidities vary in severity and hence may have different effects on the risks of mortality and healthcare costs; however, all conditions were given the same weighting in this study, given we aim to address the relationship between the number of morbidities and disease outcomes, instead of exploring specific diseases that drove the increase in risks.

## Conclusion

In conclusion, given the stronger association between multimorbidity and all-cause mortality and medical expense among younger patients, and the more economic loss due to the subsequent decreased supply of the working force, prevention and management of early-onset multimorbidity is warranted. To improve patients’ overall health, physicians should provide care for the target disease and other co-existing morbidities, ensuring a patient-centred healthcare strategy to meet their complex healthcare needs. With the multimorbidity patterns derived from a territory-wide cohort, this study can facilitate treatment planning and complication prevention strategies customised for patients of different ages and risk profiles.

## Electronic Supplementary Material

Below is the link to the electronic supplementary material.

Supplementary Material 1

## Data Availability

The datasets used and/or analysed during the current study are not available as the data custodians (the Hospital Authority of Hong Kong SAR) have not given permission for sharing due to patient confidentiality and privacy concerns. Local academic institutions, government departments, or non-governmental organizations may apply for the access to data through the Hospital Authority’s data sharing portal (https://www3.ha.org.hk/data).
