# Supplementary material for: Age-specific Multimorbidity Patterns and Burden on All-Cause Mortality and Public Direct Medical Expenditure: A Retrospective Cohort Study
Source: J Epidemiol Glob Health. 2024 Jun 13;14(3):1077–88. doi: 10.1007/s44197-024-00256-y (PMC11444029; doi:10.1007/s44197-024-00256-y)
Supplement: Supplementary file 1 — Supplementary Material 1 [file 44197_2024_256_MOESM1_ESM.docx]

**Title:** **Age-specific multimorbidity patterns and burden on all-cause mortality and public direct medical expenditure: a retrospective cohort study**

**Short title:** **Age-specific multimorbidity patterns and outcomes**

Authors: Sabrina Nan Hong^1^†, Francisco Tsz Tsun Lai, PhD^2,3^†, Boyuan Wang, MPH^1^, Edmond Pui Hang Choi, PhD^4^, Ian Chi Kei Wong, PhD^2,3^, Cindy Lo Kuen Lam, MD^1,5^, Eric Yuk Fai Wan, PhD^1,2*^

**Affiliations:**

1. Department of Family Medicine and Primary Care, School of Clinical Medicine, Li Ka Shing Faculty of Medicine, The University of Hong Kong, Hong Kong SAR, China
2. Centre for Safe Medication Practice and Research, Department of Pharmacology and Pharmacy, Li Ka Shing Faculty of Medicine, The University of Hong Kong, Hong Kong SAR, China
3. Laboratory of Data Discovery for Health (D24H), Hong Kong Science Park, Hong Kong Science and Technology Park, Hong Kong SAR, China
4. School of Nursing, Li Ka Shing Faculty of Medicine, The University of Hong Kong, Hong Kong SAR, China
5. Department of Family Medicine, the University of Hong Kong Shenzhen Hospital, The University of Hong Kong, Hong Kong SAR, China

†**Co-first author**

***Corresponding author:**

Dr Eric Yuk Fai Wan

Department of Family Medicine and Primary Care,

Li Ka Shing Faculty of Medicine,

The University of Hong Kong,

Hong Kong

Email: [yfwan@hku.hk](mailto:yfwan@hku.hk)

**Journal name:** Journal of epidemiology and global health

### Supplementary Table 1. ICPC-2 and ICD10 codes for the chronic conditions

| **Chronic conditions** | **ICD9CM** | **ICPC-2** | **ICD10** |
| --- | --- | --- | --- |
| Alcohol misuse | 265.2 291.1/291.3 291.5/291.9 303.0 303.9 305.0 357.5 425.5 535.3 571.0/571.3 980 V11.3 | P15 | E52, F10.96, F10.27, F10.951, F10.950, F10.99, G62.1, I42.6, K70.0, K70.30, K70.9, Z65.8 |
| Asthma | 493 | R96 | J45 |
| Atrial fibrillation | 427.3 | K78 | I48 |
| Cancer |  |  |  |
| Cancer lymphoma | 200/202 203.0 238.6 | B72 | C81-C96 |
| Cancer metastatic | 196/199 | B74 D74 D76 D77 L71 N74 S77 T71 U75 U76 U77 W72 X77 Y78 | C76-C80 |
| Cancer non/metastatic (breast cervical colorectal lung prostate) | 153/154 162/163 174 180 185 230.3/230.6 231.2 233.0/233.1 233.4 | D75 R84 X75 X76 Y77 | C50, C53, C18, C34, C61 |
| Chronic heart failure | 398.91 402.01 402.11 402.91 404.01 404.03 404.11 404.13 404.91 404.93 425.4/425.9 428 | K77 | I50 |
| Chronic kidney disease | 583 584 585 586 592 593.9 | U14 | N18 |
| Chronic pain | 307.80 307.89 338.0 338.2 338.4 719.41 719.45/719.47 719.49 720.0 720.2 720.9 721.0/721.4 721.6 721.8 721.9 722 723.0 723.1 723.3/723.9 724.0/724.6 724.70 724.79 724.8 724.9 729.0/729.2 729.4 729.5 | A01 | G89.21, G89.28, G89.29, G89.4, B02.22, B02.29, G03.9, G90.511, G90.512, G90.513, G90.521, G90.522, G90.523, G90.529, G90.59, G89.0, G96.12, G54.0, G54.1, G54.6, G54.8, G54.4, G56.40, G56.41, G56.42, G56.80, G56.90, G56.91, G56.92, G57.70, G57.71, G57.72 |
| Chronic pulmonary disease | 416.8 416.9 490/492 494/505 506.4 508.1 508.8 | R95 | J40-J47 |
| Cirrhosis | 571.2 571.5 571.6 456.0 456.1 456.20 456.21 567.0 567.2 567.21 567.29 567.8 567.9 572.2 572.4 789.5 (Exclude 567.81 567.82 789.51) | / | K74 |
| Dementia | 290 294.1 331.2 | P70 | F01, F02, F03 |
| Depression | 296.2 296.3 296.5 300.4 309 311 | P76 | F32 |
| Diabetes | 250 | T89/T90 | E08-E13 |
| Hypertension | 401/405 | K86 K87 | I10-I16 |
| Hypothyroidism | 240.9 243 244 246.1 246.8 | T86 | E02, E03 |
| Myocardial infarction | 410 | K75 | I21, I22, I23 |
| Peptic ulcer disease | 531.7 531.9 532.7 532.9 533.7 533.9 534.7 534.9 | D86 | K25-K28 |
| Peripheral vascular disease | 440.2 | K92 | I70.2 |
| Rheumatoid arthritis | 446.5 710.0/710.4 714.0/714.2 714.8 725 | L88 | M05, M06 |
| Schizophrenia | 295 | P72 | F20 |
| Severe constipation | 560.1 560.30 560.39 560.9 564.0 569.83 569.89 (Exclude 152/154 158 179/189 197.5/197.6 235.2 239.0 555/556 568.0 614.6 (560.9 if 789.01 789.02 789.06)) | D12 | K56.0, K56.7, K56.49, K56.600, K56.601, K56.609, K59.00, K63.1, K63.4, K63.89 |
| Stroke or transient ischemic attack | 362.3 430 431 433.01 433.11 433.21 433.31 433.81 433.91 434.01 434.11 434.91 435 436 | ­­­K90 | I60, I61, I62, I63.22, I63.139, I63.239, I63.019, I63.119, I63.219, I63.59, I63.20, I63.30, I63.40, I63.50, G45.0, G45.8, G45.1, G45.9, I67.848, I67.59 |

**Supplementary Table 2. Public health services charges**

|  | HKD ($) | USD ($) |
| --- | --- | --- |
| General ward | 4680 | 608.4 |
| Accident & Emergency Department | 990 | 128.7 |
| Specialist clinic | 1110 | 144.3 |
| General clinic | 385 | 50.1 |
| Allied health | 550 | 71.5 |

Conversion of HKD to USD is based on the exchange rate of 1:0.13.

**Supplementary Table 3. Trend of morbidity count from 2009 to 2018**

| **Year 2009** | Prevalence | |  | **Year 2014** | Prevalence | |  |
| --- | --- | --- | --- | --- | --- | --- | --- |
| No. of conditions | 0-1 | ≥2 |  | No. of conditions | 0-1 | ≥2 |  |
| Overall | 1970087 (85.98%) | 321196 (14.02%) |  | Overall | 2043953 (80.31%) | 501281 (19.69%) |  |
| Age <50 | 934581 (98.09%) | 18225 (1.91%) |  | Age <50 | 856085 (96.98%) | 26674 (3.02%) |  |
| Age 50-64 | 591447 (87.09%) | 87650 (12.91%) |  | Age 50-64 | 706618 (82.97%) | 145066 (17.03%) |  |
| Age 65-79 | 325187 (70.21%) | 137999 (29.79%) |  | Age 65-79 | 350187 (63.89%) | 197931 (36.11%) |  |
| Age ≥80 | 118872 (60.59%) | 77322 (39.41%) |  | Age ≥80 | 131063 (49.90%) | 131610 (50.10%) |  |
| **Year 2010** | Prevalence | |  | **Year 2015** | Prevalence | |  |
| No. of conditions | 0-1 | ≥2 |  | No. of conditions | 0-1 | ≥2 |  |
| Overall | 1972897 (84.45%) | 363176 (15.55%) |  | Overall | 2055778 (79.55%) | 528581 (20.45%) |  |
| Age <50 | 909440 (97.81%) | 20400 (2.19%) |  | Age <50 | 840665 (96.84%) | 27461 (3.16%) |  |
| Age 50-64 | 622172 (85.97%) | 101535 (14.03%) |  | Age 50-64 | 717524 (82.48%) | 152393 (17.52%) |  |
| Age 65-79 | 319291 (68.02%) | 150116 (31.98%) |  | Age 65-79 | 365287 (63.58%) | 209202 (36.42%) |  |
| Age ≥80 | 121994 (57.24%) | 91125 (42.76%) |  | Age ≥80 | 132302 (48.67%) | 139525 (51.33%) |  |
| **Year 2011** | Prevalence | |  | **Year 2016** | Prevalence | |  |
| No. of conditions | 0-1 | ≥2 |  | No. of conditions | 0-1 | ≥2 |  |
| Overall | 1982314 (83.24%) | 399192 (16.76%) |  | Overall | 2057833 (78.76%) | 554866 (21.24%) |  |
| Age <50 | 895580 (97.55%) | 22481 (2.45%) |  | Age <50 | 819555 (96.69%) | 28070 (3.31%) |  |
| Age 50-64 | 643427 (85.01%) | 113484 (14.99%) |  | Age 50-64 | 722407 (81.96%) | 159056 (18.04%) |  |
| Age 65-79 | 319495 (66.43%) | 161463 (33.57%) |  | Age 65-79 | 381480 (63.33%) | 220862 (36.67%) |  |
| Age ≥80 | 123812 (54.89%) | 101764 (45.11%) |  | Age ≥80 | 134391 (47.78%) | 146878 (52.22%) |  |
| **Year 2012** | Prevalence | |  | **Year 2017** | Prevalence | |  |
| No. of conditions | 0-1 | ≥2 |  | No. of conditions | 0-1 | ≥2 |  |
| Overall | 2025249 (82.29%) | 435812 (17.71%) |  | Overall | 2049849 (77.92%) | 580730 (22.08%) |  |
| Age <50 | 896389 (97.35%) | 24368 (2.65%) |  | Age <50 | 791668 (96.48%) | 28882 (3.52%) |  |
| Age 50-64 | 673824 (84.34%) | 125079 (15.66%) |  | Age 50-64 | 722457 (81.45%) | 164558 (18.55%) |  |
| Age 65-79 | 327995 (65.33%) | 174093 (34.67%) |  | Age 65-79 | 399336 (63.16%) | 232936 (36.84%) |  |
| Age ≥80 | 127041 (53.09%) | 112272 (46.91%) |  | Age ≥80 | 136388 (46.91%) | 154354 (53.09%) |  |
| **Year 2013** | Prevalence | |  | **Year 2018** | Prevalence | |  |
| No. of conditions | 0-1 | ≥2 |  | No. of conditions | 0-1 | ≥2 |  |
| Overall | 2024722 (81.14%) | 470508 (18.86%) |  | Overall | 1899162 (75.88%) | 603643 (24.12%) |  |
| Age <50 | 866315 (97.12%) | 25694 (2.88%) |  | Age <50 | 672349 (95.87%) | 28991 (4.13%) |  |
| Age 50-64 | 693428 (83.53%) | 136751 (16.47%) |  | Age 50-64 | 688502 (80.32%) | 168723 (19.68%) |  |
| Age 65-79 | 335590 (64.43%) | 185287 (35.57%) |  | Age 65-79 | 402870 (62.22%) | 244617 (37.78%) |  |
| Age ≥80 | 129389 (51.31%) | 122776 (48.69%) |  | Age ≥80 | 135441 (45.64%) | 161312 (54.36%) |  |

All parameters are expressed in frequency (percentage).

**Supplementary Table 4. Prevalence of comorbidity combinations overall and in different age groups**

| **Overall** | Percentage |  | **Age <50** | Percentage |  | **Age 50-64** | | Percentage | |
| --- | --- | --- | --- | --- | --- | --- | --- | --- | --- |
| 1. HT + DM | 51.4 |  | 1. HT + DM | 50.1 |  | 1. HT + DM | | 60.4 | |
| 2. HT + TIA | 20.9 |  | 2. HT + TIA | 11.8 |  | 2. HT + TIA | | 14.8 | |
| 3. HT + Pulmonary | 6.8 |  | 3. HT + Depression | 4.8 |  | 3. HT + Depression | | 4.1 | |
| 4. HT + Constipation | 5.5 |  | 4. HT + Asthma | 3.2 |  | 4. HT + Hypothyroidism | | 3.6 | |
| 5. TIA + DM | 5.2 |  | 5. HT + Hypothyroidism | 3.1 |  | 5. DM + TIA | | 3.5 | |
| 6. HT + Cancer | 4.9 |  | 6. HT + CKD | 2.5 |  | 6. HT + Cancer | | 3.4 | |
| 7. HT + Depression | 3.4 |  | 7. HT + CHF | 2.0 |  | 7. HT + Asthma | | 2.7 | |
| 8. HT + CHF | 3.2 |  | 8. Depression + DM | 1.8 |  | 8. HT + Constipation | | 2.3 | |
| 9. HT + Hypothyroidism | 2.9 |  | 9. HT + Cancer | 1.7 |  | 9. HT + Pulmonary | | 1.8 | |
| 10. HT + Asthma | 2.6 |  | 10. Alcohol + Cirrhosis | 1.6 |  | 10. HT + Alcohol | | 1.6 | |
|  |  |  |  |  |  |  |  | |  |
| **Age 65-79** | Percentage |  | **Age ≥80** | Percentage |  |  |  | |  |
| 1. HT + DM | 54.5 |  | 1. HT + DM | 35.9 |  |  |  | |  |
| 2. HT + TIA | 21.7 |  | 2. HT + TIA | 29.1 |  |  |  | |  |
| 3. HT + Pulmonary | 7.3 |  | 3. HT + Pulmonary | 13.7 |  |  |  | |  |
| 4. DM + TIA | 6.3 |  | 4. HT + Constipation | 10.2 |  |  |  | |  |
| 5. HT + Cancer | 5.7 |  | 5. HT + CHF | 7.2 |  |  |  | |  |
| 6. HT + Constipation | 5.5 |  | 6. DM + TIA | 6.5 |  |  |  | |  |
| 7. HT + Depression | 2.8 |  | 7. HT + Cancer | 6.1 |  |  |  | |  |
| 8. HT + Hypothyroidism | 2.7 |  | 8. HT + AF | 4.8 |  |  |  | |  |
| 9. HT + Asthma | 2.7 |  | 9. HT + Dementia | 4.3 |  |  |  | |  |
| 10. HT + AF | 2.5 |  | 10. HT + Depression | 3.3 |  |  |  | |  |

Percentage of patients with the corresponding morbidity combinations based on patients with multimorbidity.

**Supplementary Table 5. Prevalence of comorbidity combinations overall and in different age groups**

| **Overall** | Percentage |  | **Age <50** | Percentage |  | **Age 50-64** | Percentage |
| --- | --- | --- | --- | --- | --- | --- | --- |
| 1. DM + TIA | 5.2 |  | 1. DM + Depression | 1.8 |  | 1. DM + TIA | 3.5 |
| 2. DM + Constipation | 1.8 |  | 2. Alcohol + Cirrhosis | 1.6 |  | 2. DM + Depression | 1.5 |
| 3. DM + Cancer | 1.5 |  | 3. DM + TIA | 1.5 |  | 3. DM + Cancer | 1.2 |
| 4. DM + Pulmonary | 1.4 |  | 4. Constipation + Depression | 1.4 |  | 4. DM + Constipation | 1.1 |
| 5. Pulmonary + TIA | 1.4 |  | 5. Asthma + Depression | 1.4 |  | 5. DM + Hypothyroidism | 1.1 |
| 6. DM + Depression | 1.1 |  | 6. DM + Asthma | 1.2 |  | 6. Alcohol + Cirrhosis | 1.1 |
| 7. Constipation + TIA | 1.1 |  | 7. DM + Schizophrenia | 1.1 |  | 7. DM + Asthma | 0.9 |
| 8. DM + Hypothyroidism | 0.9 |  | 8. DM + Hypothyroidism | 1.1 |  | 8. Depression + TIA | 0.6 |
| 9. DM + Asthma | 0.8 |  | 9. Depression + Hypothyroidism | 1.0 |  | 9. DM + Alcohol | 0.6 |
| 10. Depression + TIA | 0.8 |  | 10. DM + Constipation | 1.0 |  | 10. Asthma + Pulmonary | 0.5 |
|  |  |  |  |  |  |  |  |
| **Age 65-79** | Percentage |  | **Age ≥80** | Percentage |  |  |  |
| 1. DM + Constipation | 6.3 |  | 1. DM + TIA | 6.5 |  |  |  |
| 2. DM + TIA | 1.9 |  | 2. Pulmonary + TIA | 3.0 |  |  |  |
| 3. DM + Cancer | 1.8 |  | 3. DM + Constipation | 2.5 |  |  |  |
| 4. DM + Pulmonary | 1.7 |  | 4. DM + Pulmonary | 2.3 |  |  |  |
| 5. Pulmonary + TIA | 1.3 |  | 5. Constipation + TIA | 2.2 |  |  |  |
| 6. Constipation + TIA | 1.1 |  | 6. Dementia + TIA | 1.7 |  |  |  |
| 7. DM + Depression | 0.9 |  | 7. DM + CHF | 1.6 |  |  |  |
| 8. DM + Hypothyroidism | 0.9 |  | 8. AF + TIA | 1.5 |  |  |  |
| 9. DM + Asthma | 0.9 |  | 9. Constipation + Pulmonary | 1.5 |  |  |  |
| 10. DM + AF | 0.9 |  | 10. CHF + Pulmonary | 1.5 |  |  |  |

Percentage of patients with the corresponding morbidity combinations based on patients with multimorbidity.

**Supplementary Figure 1. Chord diagrams of the relative frequencies of morbidity combinations of the five most prevalent diseases in each age group.**

1. **Age < 50**

**
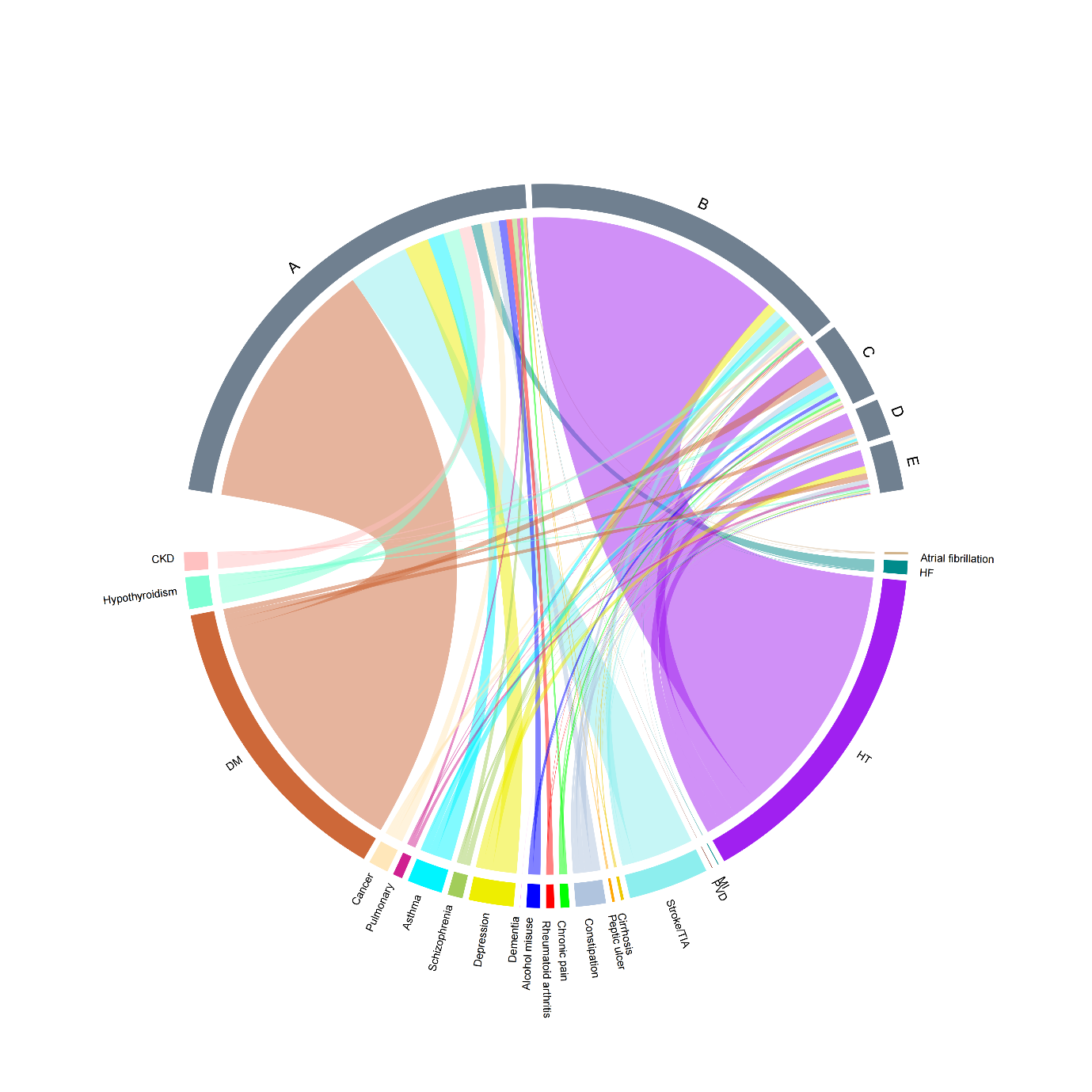
**

A: HT

B: DM

C: Depression

D: Hypothyroidism

E: Asthma

1. **Age 50-64**

**
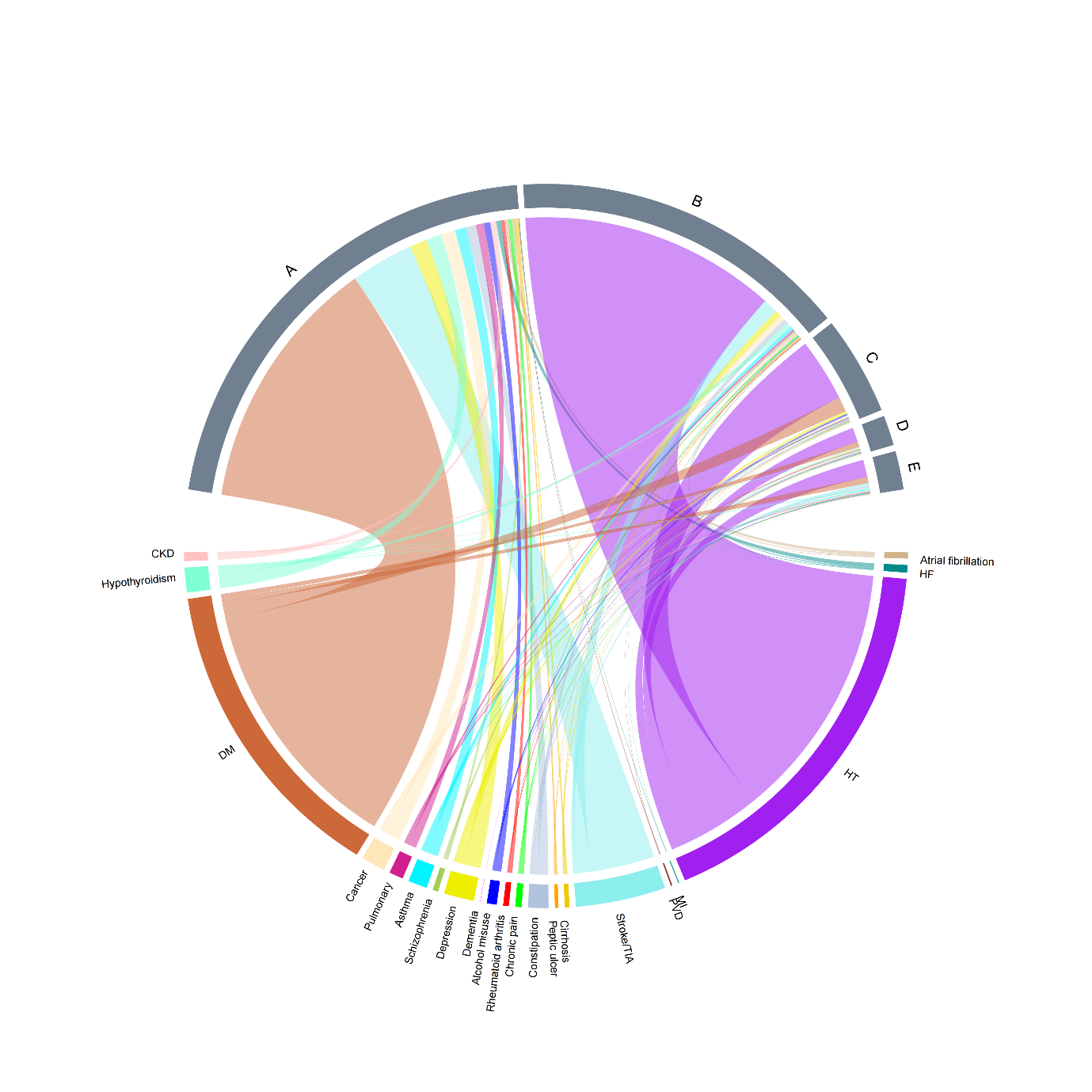
**

A: HT

B: DM

C: Stroke/TIA

D: Cancer

E: Depression

A: HT

B: DM

C: Stroke/TIA

D: Pulmonary

E: Cancer

1. **Age 65-79**

**
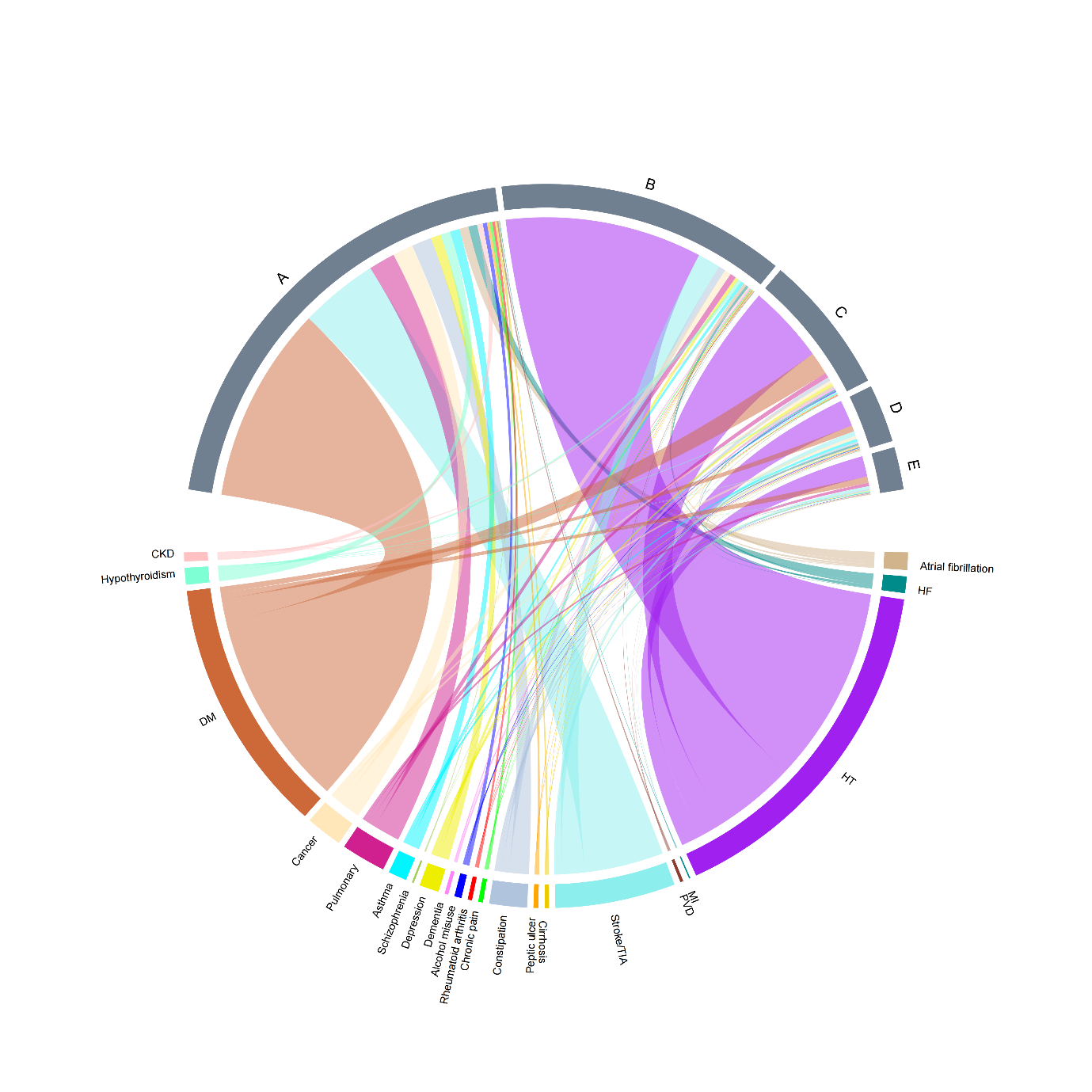
**

A: HT

B: DM

C: Stroke/TIA

D: Pulmonary

E: Cancer

**(D) Age ≥ 80**

**
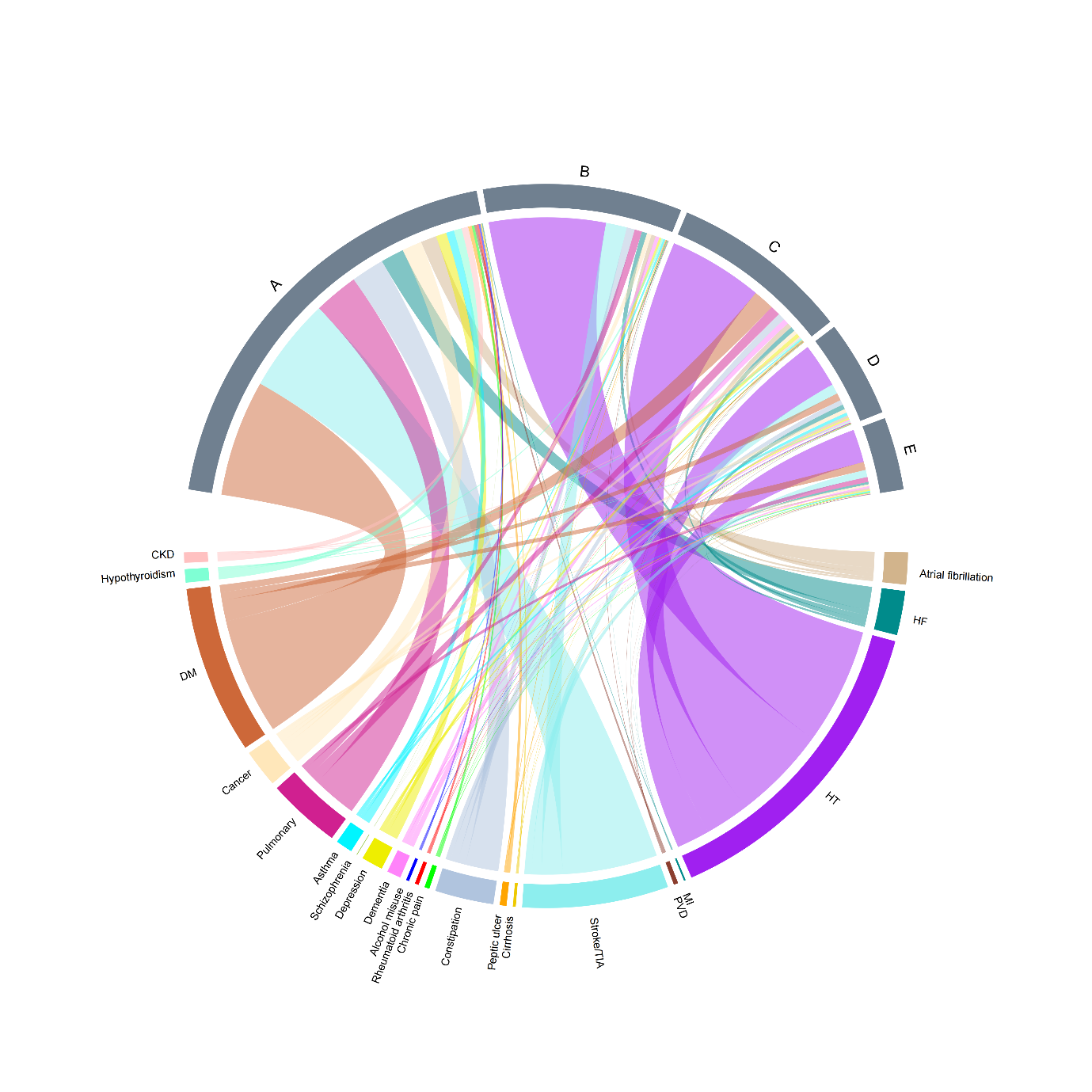
**

A: HT

B: DM

C: Stroke/TIA

D: Pulmonary

E: Constipation

HF: Chronic heart failure; HT: Hypertension; MI: Myocardial infarction; PVD: Peripheral vascular disease; Stroke/TIA: Stroke or transient ischemic attack; Constipation: Severe constipation; Pulmonary: Chronic pulmonary disease; DM: Diabetes; CKD: Chronic kidney disease.
